# Pioneering Metabolomic Studies on *Diaporthe eres* Species Complex from Fruit Trees in the South-Eastern Poland

**DOI:** 10.3390/molecules28031175

**Published:** 2023-01-25

**Authors:** Barbara Abramczyk, Łukasz Pecio, Solomiia Kozachok, Mariusz Kowalczyk, Anna Marzec-Grządziel, Ewa Król, Anna Gałązka, Wiesław Oleszek

**Affiliations:** 1 Department of Agricultural Microbiology, Institute of Soil Science and Plant Cultivation—State Research Institute, Czartoryskich 8, 24-100 Puławy, Poland; 2 Department of Biochemistry and Crop Quality, Institute of Soil Science and Plant Cultivation—State Research Institute, Czartoryskich 8, 24-100 Puławy, Poland; 3 Department of Natural Products Chemistry, Medical University of Lublin, 20-093 Lublin, Poland; 4 Department of Plant Protection, University of Life Sciences in Lublin, Leszczyńskiego 7, 20-069 Lublin, Poland

**Keywords:** *Diaporthe eres* species complex, fruit plants, chemotaxonomic markers, metabolite profiling

## Abstract

Fungi from the genus *Diaporthe* have been reported as plant pathogens, endophytes, and saprophytes on a wide range of host plants worldwide. Their precise identification is problematic since many *Diaporthe* species can colonize a single host plant, whereas the same *Diaporthe* species can inhabit many hosts. Recently, *Diaporthe* has been proven to be a rich source of bioactive secondary metabolites. In our initial study, 40 *Diaporthe* isolates were analyzed for their metabolite production. A total of 153 compounds were identified based on their spectroscopic properties—Ultraviolet-visible and mass spectrometry. From these, 43 fungal metabolites were recognized as potential chemotaxonomic markers, mostly belonging to the drimane sesquiterpenoid-phthalide hybrid class. This group included mainly phytotoxic compounds such as cyclopaldic acid, altiloxin A, B, and their derivatives. To the best of our knowledge, this is the first report on the metabolomic studies on *Diaporthe eres* species complex from fruit trees in the South-Eastern Poland. The results from our study may provide the basis for the future research on the isolation of identified metabolites and on their bioactive potential for agricultural applications as biopesticides or biofertilizers.

## 1. Introduction

The genus *Diaporthe* Nitschke belongs to the family Diaporthaceae, (Diaporthales, Diaporthomycetidae, Sordariomycetes, Pezizomycotina, Ascomycota; (MycoBank. 2022; Species Fungorum. 2022; accessed on 21 December 2022), with the anamorph known as *Phomopsis*. According to the implementation of “one fungus one name (1F:1N) nomen- clature”, Diaporthe has been adopted over Phomopsis because it was first introduced as this, is encountered commonly in the literature, and represents most species [1]. *Diaporthe* are present as plant pathogens, endophytes, and saprophytes in a wide range of hosts worldwide [2,3,4,5]. Some pathogenic *Diaporthe* are responsible for several serious diseases of economically important crops, including fruit plants [4,6,7]. Both sexual and asexual morphs of *Diaporthe* have been associated with cankers, shoot diebacks, bud and shoot blights, and leave spots of peach caused by *Phomopsis amygdali* [8], apple by *P. mali* [9], pear by *D. eres* [10], plum by *D. perniciosa* [11], grape by *P. viticola*, *P. fukushii*, *D. eres* [12,13], blueberry by *D. australafricana*, *D. ambigua*, *D. neotheicola*, *D. passiflorae* [14], and many others. Currently, 1177 names of *Diaporthe* and 984 of *Phomopsis* are listed in Index Fungorum (http://www.indexfungorum.org/; accessed on 20 September 2022). Identification of *Diaporthe* species is complicated and was initially based on morphological features, cultural characteristics, and host affiliation leading to a proliferation of names based on the hosts from which they were isolated [15]. It has been observed that the same *Diaporthe* species colonizes different hosts, and the co-occurrence of different species is commonly reported in the same host [4,16,17,18,19]. Thus, the identification and description of species based on host association are unreliable within *Diaporthe* [3,20,21]. Moreover, the identification of *Diaporthe* based only on morphological features such as the size and shape of ascomata [2] and conidiomata [16] also proved insufficient due to their variability under changing environmental conditions [3]. Currently, the taxonomy of *Diaporthe* is actively changing, with numerous species being described each year, primarily based on molecular data combined with morphological characterization and host associations [3,10,20,21,22,23,24].

In recent years the genus *Diaporthe* has been widely used in secondary metabolite study due to their production of a variety of unique low- and high-molecular-weight metabolites with different bioactivities which were recently summarized in the extensive review by Xu et al. [25]. Researchers focused mainly on the endophytic species of *Diaporthe*, which is reported as one of the most frequently isolated genera among endophytic fungi. Probably the same compounds can be produced by endophytic, saprotrophic, and pathogenic species [26]. Over the past decade, 335 bioactive secondary metabolites have been obtained from known *Diaporthe* species and from those for which only a generic name has been assigned. [25,27,28] Among bioactive compounds 246 were isolated from *Phomopsis*, 106 from *Diaporthe*, and 17 from both species [25,29]. The metabolites produced by this genus include terpenoids, steroids, macrolides, ten-membered lactones, alkaloids, flavonoids, fatty acids, and polyketides, being the main structural type [25].

Although endophytic *Diaporthe* species have been extensively screened in bioassays for metabolite production, no such information is available for fungi belonging to *D. eres* species complex isolated from fruit trees in Poland. The literature indicates that the same species of the genus *Diaporthe* can occur on one or different hosts with different lifestyles [2]. Some *Diaporthe* species described as endophytes include latent phytopathogens, which asymptomatically colonize various host plants [30]. An example is *D. eres*, which as a pathogen infects many crops, including orchards, and is often the main cause of serious economic losses worldwide [3,20,21,31]. In Poland, however, the endophytic form of *D. eres* on *Prunus domestica* was recorded [32]. In the era of global warming and climate change, we must remember that many species may switch their lifestyles or spread into new regions, where they will come into contact with new potential hosts and will become a dangerous cause of diseases [33,34]. Therefore, the additional data such as metabolite profiles of such important fungi like *Diaporthe* may be crucial to understand their pathogenicity and switching life mode triggers in future research. Since *Diaporthe* species are a valuable source of bioactive metabolites, it would be worthwhile to further explore the genus for novel compounds that have a biotechnological potential.

The main objective of our study was metabolite profiling of *Diaporthe* isolates from various orchard plants of south-eastern Poland. To fully delineate the secondary metabolite profile of any fungus is an ambitious undertaking. Thus, this work is just an initial step toward the further exploration of the novel compounds from *Diaporthe* and their agricultural or pharmaceutical bioactivities.

## 2. Results and Discussion

### 2.1. ITS-Based Fungal Identification

The sequences of the ITS regions were used to identify *Diaporthe* strains. Closely related species have been received by comparing the obtained sequence data with the NCBI database (https://www.ncbi.nlm.nih.gov; accessed on 19 December 2022). The results indicated that 34 of the tested strains were closely related to *D. eres* species complex with 100% of similarity, 5 strains with 99.8%, and one strain with 99.6% of similarity (Table 1).

### 2.2. Chemical Characterization of Fungal Metabolites

Our study revealed that the *D. eres* species complex isolated from fruit trees in south-eastern Poland showed high biodiversity in the secondary metabolite production. A total of 153 compounds were found as a result of screening of forty isolates belonging to the *Diaporthe eres* species complex, based on their spectroscopic properties—UV-Vis and mass spectrometry (Table 2). The identified metabolites mainly included polyketides, pyrones, fatty acids/oxylipins, chromones, sesquiterpenoids, phthalides, and numerous derivatives and hybrids belonging to the preceding groups of compounds. The metabolite profile of the studied isolates belonging to the *Diaporthe eres* species complex is unique and most of the detected compounds have not been described for *Diaporthe* species before. Furthermore, as far as we know, our research on the characterization of the metabolite profile of *D. eres* species complex isolated from orchard plants is pioneering and has not been conducted in Europe or other parts of the world before. There are few publications on *Diaporthe (=Phomopsis)* from fruit plants, but they focus mainly either on *Diaporthe* from the one host plant or on the selected group of metabolites produced by *Diaporthe* [35,36].

#### 2.2.1. Polyketides

**Figure 1:**
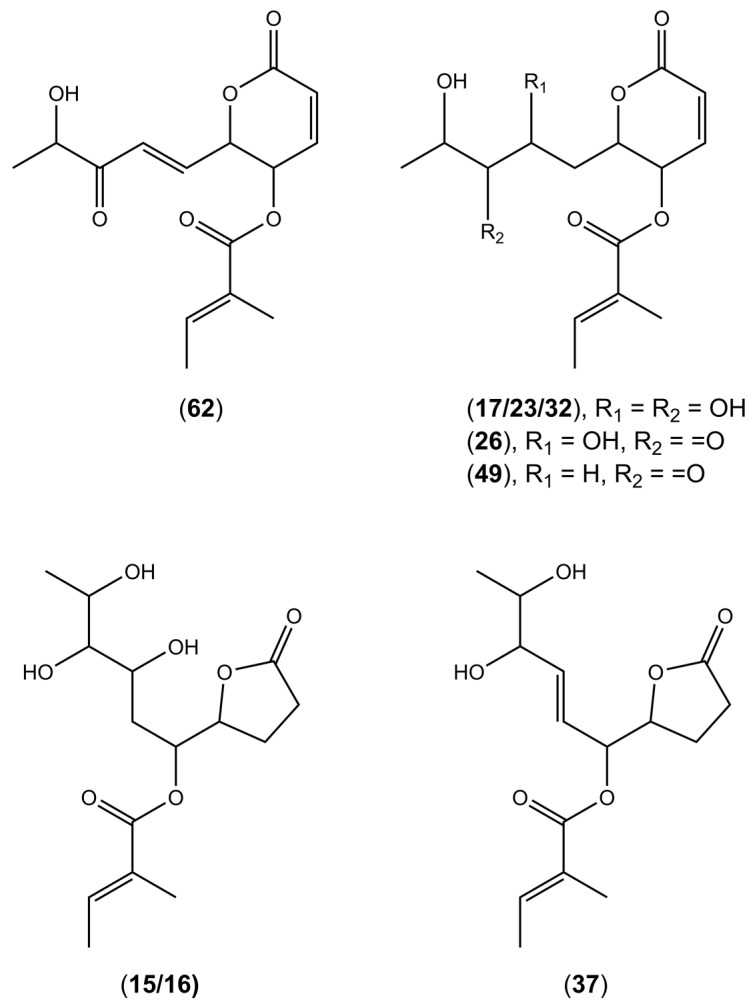
Putative structures of polyketides found in the tested *Diaporthe* isolates.

The UHPLC-HRESIMS analysis of extracts from *Diaporthe* isolates led to the annotation of several compounds from the polyketide group—pyranones such as, dihydrohydroxyphomopsolide B isomers I–III (**17**, **23**, **32**), dihydrophomopsolide A (**49**), dihydrohydroxyphomopsolide A (**26**), and furanones such as phomopsolidone B (**37**) and dihydrohydroxyphomopsolidone B isomers I and II (**15**, **16**) (Figure 1). These compounds were tentatively identified based on the high-resolution mass of the precursor ions and the fragments generated via common fragmentation pathways in positive ionization mode. Namely, the loss of one or two water molecules (−18 Da or 36 Da), followed by the loss of a tiglic acid (2-methylbut-2-enoic acid) residue (-C_5_H_8_O_2_), giving intense fragment ions with *m/z* 179 or 197 for compounds **17**, **23**, and **32** ion at *m/z* 177 for compounds **26** and **49**, and *m/z* 181 for compounds **15**, **16**, and **37**, was observed (Table 2). Phomopsolides are common secondary metabolites derived from *Diaporthe* [25]. They were initially isolated from *Phomopsis oblonga*, a fungus that provided some protection against elm bark beetle infestations [37]. They have been proved for their antibacterial activity against *Staphylococcus aureus* [38]. Moreover, phomopsolide A/C (**62**), from the endophytic *Diaporthe* sp. AC1 from *Artemisia argyi*, was proved to inhibit the growth of *Fusarium graminearum*, *F. moniliforme*, *Botrytis cinerea*, and *Verticillium dahliae*, indicating that the compound may have a broad spectrum of antifungal activity [29].

#### 2.2.2. Pyrones

**Figure 2:**
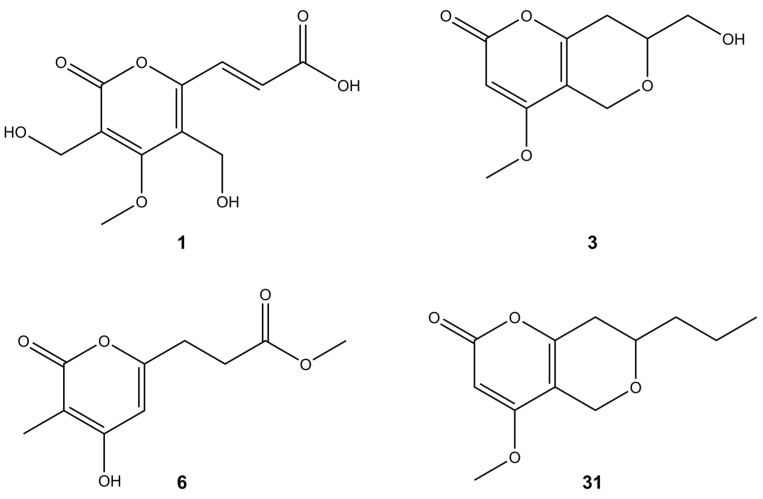
Putative structures of pyrones found in the tested *Diaporthe* isolates.

Pyrones represent a class of oxygen-based heterocyclic compounds that naturally occur in two isomeric forms as either 2-pyrone (α-pyrone) or 4-pyrone (γ-pyrone). The number 2/4 is assigned based on the position of the carbonyl group relative to the oxygen atom within the ring system [39]. In our study, *Diaporthe* spp. isolated from fruit trees produced phomopsinone A (**31**) and pyrenocine P (**3**) (Figure 2), which belong to the α-pyrones. Their fragmentation spectra showed mainly water (−18 Da) and/or CO losses (−28 Da). However, characteristic UV maxima at around 280 nm indicated α-pyrone structures (Table 2). Previously, phomopsinone A and pyrenocine J-M have been isolated from the endophytic fungus *Phomopsis* sp. and have shown antifungal, antibacterial, and antialgal activity [27,28]. Phomopsinone A showed very strong antifungal activity against *Botrytis cinerea*, *Pyricularia oryzae*, and *Septoria tritici*. Pyrenocine J-M had strong antibacterial activity especially against the gram-negative bacterium *E. coli*, since gram-negative bacteria are usually difficult to inhibit. Similarly, all mentioned compounds showed algicidal activity against *Chlorella fusca* [27,28].

The studied *Diaporthe* isolates, apart from the metabolite characteristics for the genus *Diaporthe*, also produced several new bioactive compounds usually present in the other species of fungi but not in *Diaporthe* [39]. For example, islandic acid-II (**1**), originally isolated from *Penicillium islandicum*, in the literature was reported as showing the complete growth inhibition of Yoshida sarcoma tumor cells [40]. Another compound produced by the tested *Diaporthe* isolates was scirpyrone K (**6**) (Figure 2). Its fragmentation pathway was very similar to that of compound **3**. Previously, it had been isolated from a marine fungus identified as *Phialocephala* sp. strain FL30r. This compound exhibited weak radical scavenging activity with no cytotoxic activities reported [41].

#### 2.2.3. Oxylipins

Oxylipins constitute a large family of oxidized fatty acids and their derivatives. Bioactive lipid production is widespread among many organisms including filamentous fungi [42]. In many cases, oxylipins have a role in both organismal development and communication with the host on a cellular basis [43,44]. The literature showed that fungal oxylipins are involved in influencing processes in infected host tissues, presumably by mimicking endogenous signal molecules [45,46]. Fungi have the ability to use the host plant’s oxylipin to achieve their own benefits. For example, by increasing the production of toxins, they improve their virulence [45,46], and by increasing sporulation they can accelerate reproduction in the tissues of the host plant [47]. Additional functions of fungal oxypilins have also been reported. They are related to fungal development regulation, metabolism, and host-pathogen interaction [42,48,49]. The synthesis of oxylipins proceeds due to substrates released by phospholipids and acylglycerides such as: oleic, linoleic, linolenic, and arachidonic acids [50,51]. Various reactions occurring in an oxidizing environment, in combination with enzymatic activity, contribute to the formation of various oxylipins from a given fatty acid. [52]. In our study we have tentatively identified thirty-seven oxylipins of predominantly C_18_ chain (Table 2); among them, trihydroxyoctadecenoic acid isomers I-VII (**56**, **69**, **71**, **74**, **81**, **83**, **85**) have been found in the tested *Diaporthe* isolates. It should be mentioned that the differences in fragmentation patterns between structural isomers were minimal and did not allow us to determine the position of double bonds or hydroxyl groups in the analyzed compounds. Previously, similar metabolites have been produced in the tubers of taro (*Colocasia antiquorum*) as a defense response to inoculation with black rot fungus (*Ceratocystis fimbriata*) [53]. They were isolated for the first time from the Chinese truffle *Tuber indicum* [54]. It has been proven, for example, that (9*S*,12*S*,13*S*)-tri-hydroxyoctadeca-10*E*-enoic acid had antifungal activities against *Magnaporthe grisea* causing rice blast disease [55], and (13*S*)-hydroxy-9,11-octadecadienoic acid had nematocidal properties [56].

#### 2.2.4. Chromones

**Figure 3:**
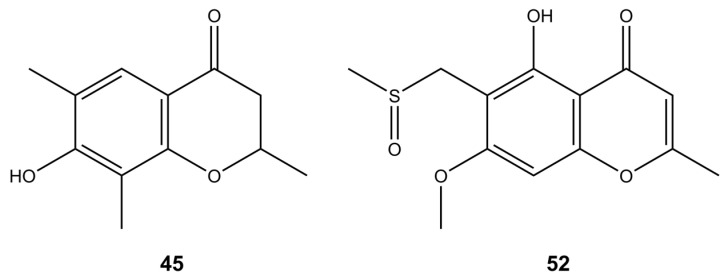
Putative structures of chromones found in the tested *Diaporthe* isolates.

Chromones are naturally occurring phenolic derivatives of chromone (1,4-benzopyrone or 4H-chromen-4-one) and are isomers of coumarin. They are produced abundantly by many genera of plants, being a part of a normal healthy diet and by fungi. This class of compounds is mainly associated with antioxidant, antimicrobial, anticancer, and anti-inflammatory activities [57]. In our study, *Diaporthe* spp. produced phomochromone A (**45**) (Figure 3), which can exhibit an antifungal, antibacterial, and algicidal activities, which is supported by the literature. For example, two new chromones, phomochromone A and B, have been isolated from the endophytic fungus *Phomopsis* spp. from *Cistus monspeliensis* which showed good antifungal, antibacterial, and algicidal properties towards *Septoria tritici*, *Microbotryum violaceum*, *Botrytis cinerea*, *E. coli, Bacillus megaterium*, and *Chlorella fusca* [58]. Amycolachromone E (**52**) (Figure 3) and the series of other chromone derivatives were isolated from the deep-sea marine actinomycete *Amycolatopsis* sp. [59].

#### 2.2.5. Sesquiterpenoids

**Figure 4:**
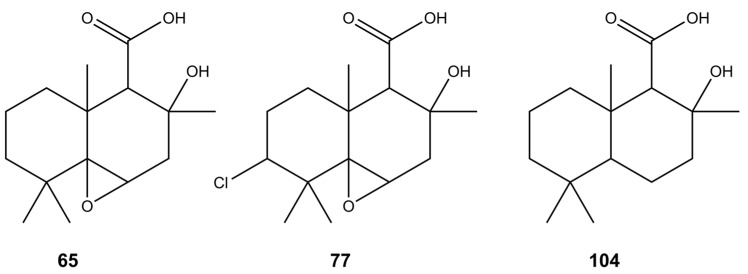
Putative structures of sesquiterpenoids found in the tested *Diaporthe* isolates.

Drimane-type sesquiterpenoids are a large group of compounds that have been found in plants and fungi, exhibiting various biological activities [60,61]. During the research conducted by Zang et al. [62] and Chen et al. [63], a variety of new drimane-type metabolites, including diaporols B–I (**104**), Q, and R, have been isolated from the mangrove endophytic *Diaporthe* sp. [62,63]. Furthermore, two drimane-type sesquiterpenoids, named altiloxins A (**65**) and B (**77**) (Figure 4), showing phytotoxic activity on the lettuce seedlings were obtained from *Phoma asparagi* [64]. Considering the fragmentation spectra of compounds **65**, **77**, and **104**, in the *Diaporthe* isolates studied, we determined the presence of a number of their derivatives—dihydro-altiloxin A (**54**), dihydro-altiloxin B (**72**), hydroxy-altiloxin A isomers I and II (**19**, **22**), hydroxy-altiloxin B isomers I and II (**33**, **50**), and deoxy-altiloxin A (**102**).

#### 2.2.6. Phthalides

Phthalides are natural substances used in traditional medicine in Asia, Europe, and North America, which can be found both in plants and fungi [65,66,67,68,69]. In our study *Diaporthe* spp. produced the convolvulanic acid A isomers I–II (**9**,**13**), which was previously reported from *Phomopsis convolvulus*, a host-specific pathogen of field bindweed (*Convolvulus arvensis*) [66]. This metabolite showed phytotoxic activity against *C. arvensis*, proving that it could be used as an herbicide to control this weed effectively [66].

#### 2.2.7. Hybrid Compounds

**Figure 5:**
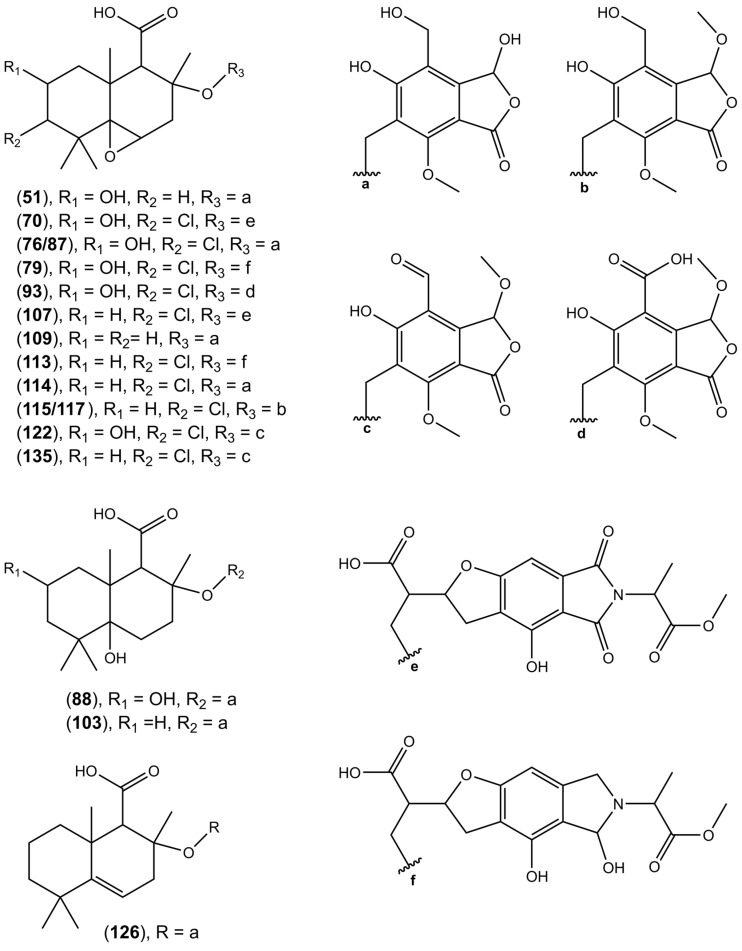
Putative structures of hybrid sesquiterpenoids-phthalides found in the tested *Diaporthe* isolates.

HRESIMS analysis revealed a molecular formula of C_27_H_33_ClO_10_ ([M − H]^−^ at *m/z* 551.1692) for compound **135**, suggesting close structural analogy to pestalotiopene A [68]. The structural similarity of both compounds was further corroborated by detecting the same mass fragment at *m*/*z* 301.1206 with the characteristic chlorine isotope splitting, corresponding to the altiloxin B part of pestalotiopene A. Previously, drimane sesquiterpene-cyclopaldic acids hybrids, pestalotiopens A and B, were isolated from the mangrove-derived fungus *Pestalotiopsis* sp. obtained from leaves of the Chinese mangrove *Rhizophora mucronate* [68]. Pestalotiopen A (**135**), an altiloxin B—*O*-methylcyclopaldic acid hybrid, showed moderate antibacterial activity against *Enterococcus faecalis* [68]. Cyclopaldic acid was also produced by *Seiridium cupressi,* the pathogen of a canker disease of cypress, showing phytotoxic and antifungal activity [67], and by *Coccomyces strobi* isolated from needles of *Pinus strobus*, showing moderate growth inhibition of *Microbotryum violaceum* (=*Ustilago violacea*) and weak antibiotic activity against *Bacillus subtilis*, with no inhibition observed against E. coli at the highest tested concentration [69]. In the search for natural products as an alternative to synthetic pesticides, cyclopaldic acid has been reported to possess insecticidal [70], fungicidal [71], as well as herbicidal [72] activities. Recently, Samperna et al. [73], during the investigation of the effects of cyclopaldic acid in *Arabidopsis thaliana* plants and protoplasts, showed that this metabolite induced leaf chlorosis, ion leakage, membrane-lipid peroxidation, hydrogen peroxide production, and inhibited root proton extrusion in vivo and plasma membrane H^+^-ATPase activity in vitro. In our study, we report the presence of over twenty-five compounds, ethers of altiloxin A and its derivatives with cyclopolic acid (**51**, **88**, **103**, **109**, **126**, and **127**), and ethers of altiloxin B and its derivatives with either (iso)cyclopaldic acid, cyclopolic acid, or salfredins A7/C3 (**57**, **63**, **66**, **70**, **76**, **79**, **87**, **90**, **91**, **93**, **95**, **98**, **101**, **106**, **107**, **112–115**, **117**, **122**, and **135**) (Figure 5). The identity of these compounds was tentatively established by the similarity of fragmentation spectra to those of compound **135** (Table 2).

### 2.3. Metabolite-Based Chemotaxonomy

**Figure 6:**
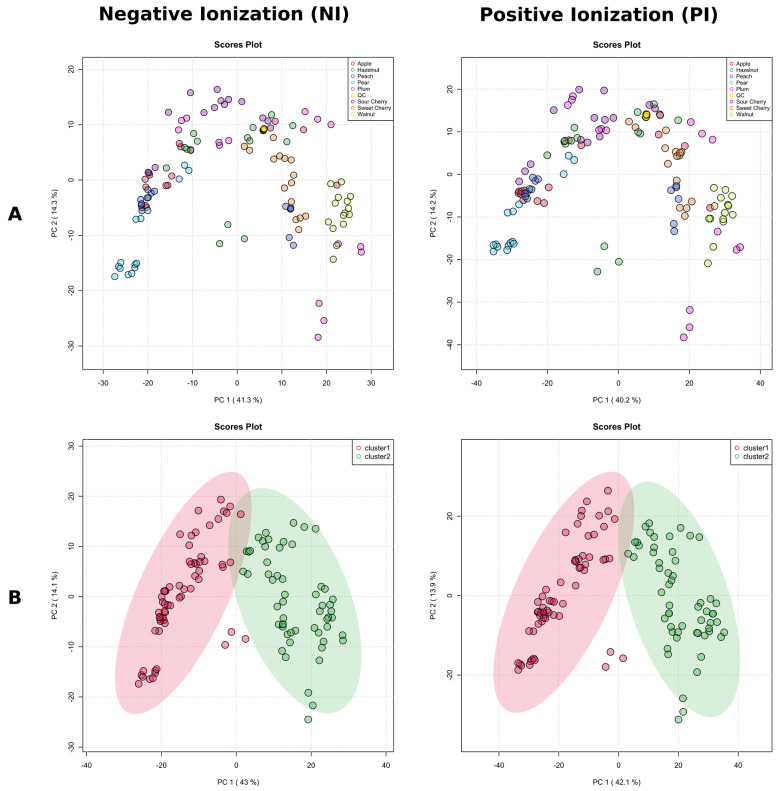
The score plots of principal component analysis (PCA) in negative ionization mode and positive ionization mode LC-MS data of the tested *Diaporthe* isolates, where each point represents a single isolate. (**A**) PCA colored by the host plant. (**B**) Isolates in the PCA are colored by *k*–means clustering cluster assignments from negative ionization mode; the elliptic areas represent the 95% confidence regions.

As a preliminary step in multivariate statistical analysis, PCA analysis provided an unsupervised overview of LC-MS fingerprints obtained in both ionization modes (NI and PI). Both NI and PI PCA score plots revealed a close clustering of the QC samples (Figure 6A), indicating that the separation, observed between fungal isolates into two distinct chemotypes was mainly due to biological reasons.

**Figure 7:**
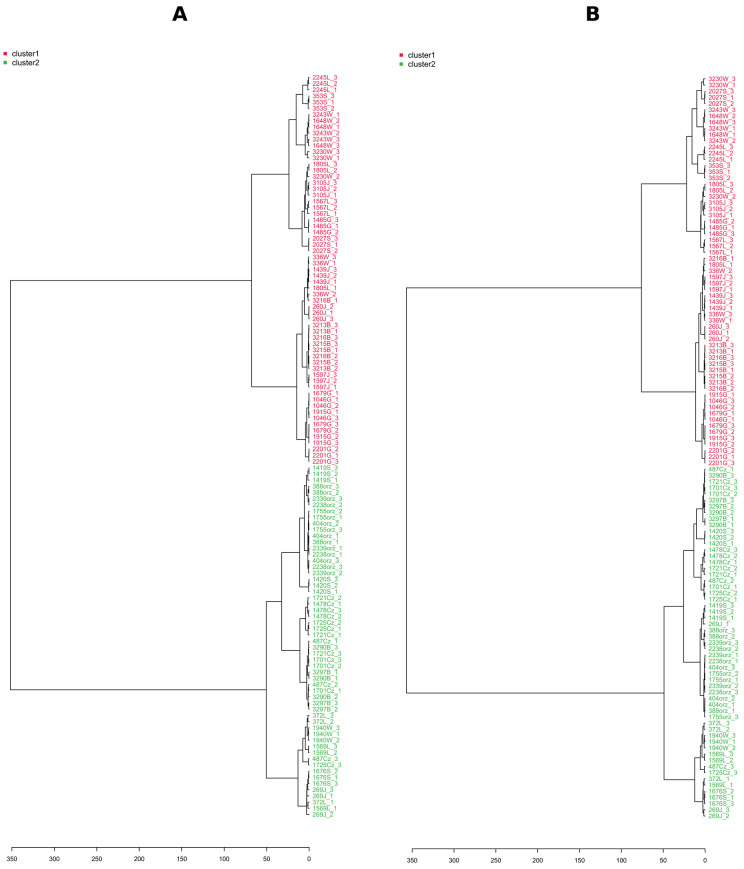
LC-MS-based hierarchical cluster analyses (HCA, Pearson distance, and Ward’s linkage rule) show the tested *Diaporthe* isolates following differentiation in negative ionization mode (**A**) and positive ionization mode (**B**). Letters in isolate names refer to the host plant: J = Apple; L = Hazelnut; B = Peach; G = Pear; S = Plum; W = Sour Cherry; Cz = Sweet Cherry; and orz = Walnut. Numbers 1, 2, and 3 after the underscore refer to individual biological repetitions.

To avoid biased group assignment of the PCA plots, samples were statistically assigned into 2 clusters (chemotypes) based on the *k*–means clustering algorithm in NI mode, and the groups generated by the *k*–means clustering algorithm in the negative mode were assigned to the positive mode (Figure 6B). The clustering of the data was easily visualized in both ionization modes, and confirmed by clusters obtained separately by HCA (Figure 7A,B). The first five PCs explained 75.1% of the variance in NI and 75.4% in PI modes, and 57.1% of the total variance was projected in the first two PCs in NI, while 56.0% in PI, which suggested the similar quality of data obtained in both ionization modes. Indeed, the PCA score plots showed similar patterns with specific host plants (understood as metadata) grouped together (pear, sweet cherry, and walnut), while the rest were much more dispersed, and there were no clear associations between the metadata and the groups in the PCA. We decided to use NI mode for further work due to the lower complexity of LC-MS data (high amount of in-source collision-induced dissociation in PI).

**Figure 8:**
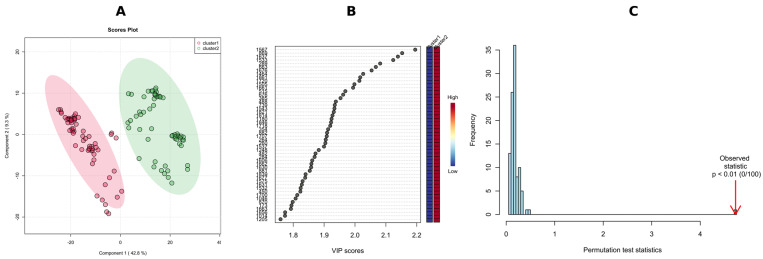
PLS-DA score plots of clusters in NI-mode-based *k*–means clustering of the tested *Diaporthe* isolates; the elliptic areas represent the 95% confidence regions (**A**); the top 50 features ranked based on scores of VIP, features are numbered based on MS-DIAL ID (see Table 2) (**B**); and permutation test results of the PLS-DA model (statistical test: separation distance (B/W)), the number of permutations set at 100 (**C**).

To validate the *k*–means/HCA model and to identify the features responsible for the classification, we performed a supervised PLS-DA analysis, and overall, 52.1% of the total variance was displayed on the first two principal component axes of the PLS-DA score plot (Figure 8A), with R^2^X = 0.946 and Q^2^ = 0.924 calculated from the first three components via a 10-fold cross-validation method, with Q^2^ as the measured performance. Since PLS-DA tends to overfit data, the model was validated to understand whether the separation is statistically significant or is due to random noise. This hypothesis was tested using the permutation test—separation distance (B/W), with 100 permutations with observed statistics having a *p* < 0.01 (Figure 8C).

A *p*-value below 0.01 in 100 permutations means that not even once (<0.01 × 100) did the permutated data yield a better performance (higher B/W) than the original label, suggesting the significant difference between these two clusters.

**Figure 9:**
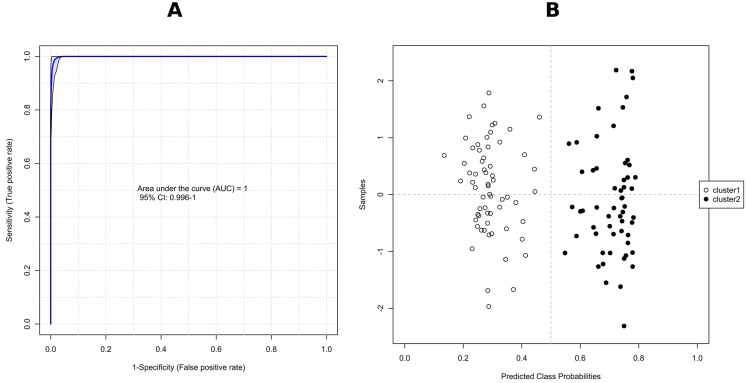
ROC curve for combined biomarker models (set of 43 metabolites); 100 cross-validations were performed, and the results were averaged to generate the plot (**A**); The average of predicted class probabilities of each sample across the 100 cross-validations. As the algorithm uses a balanced sub-sampling approach, the classification boundary is located at the center (x = 0.5, the dotted line). The corresponding confusion matrix showed that all isolates were correctly classified in all cases (**B**).

Potential variables to separate clusters 1 and 2 in the dendrogram were identified as potential biomarkers using VIP values which estimate the importance of each variable in the projection used in a PLS-DA model. The greater-than-one rule is usually considered for detecting the descriptors with the greatest importance in the projection. However, we decided, due to a large number of significant metabolites (>300), to use VIP scores > 1.8 (Figure 8B). The peak intensity ratios were also subjected to an unpaired non-parametric test (Wilcoxon rank-sum test, also known as the Mann–Whitney U test) within MetaboAnalyst, and false discovery rates (FDR < 0.05) were calculated to discover if those features are significantly different between cluster 1 and 2. A large fold change (FC > 10) between the two putative chemotypes was also considered a selection criterion, with FC > 100 indicating the presence/absence of the feature in question. As a result, 43 features meeting these conditions (VIP = 2.20–1.81, FDR adj. *p*-value = 3.23 × 10^−19^–4.71 × 10^−18^, FC = 348–19) were selected (Table 3) for receiver operating characteristic (ROC) analysis in order to assess their potential as chemotaxonomical biomarkers. ROC curves are used to evaluate classification and prediction models in bioinformatics. They are often summarized in a single metric known as area under the curve (AUC), where AUC = 1.0 indicates an excellent classifier and AUC = 0.5 means the classifier has no practical utility [74]. In this regard, we calculated the AUC for each selected candidate biomarker, and the AUC values obtained ranged from 0.972 to 1.000 (Table 3). Furthermore, to consider factors other than genetics, i.e., host plant, year of strain isolation or storage time, a combination of multiple individual markers must be considered into a single multivariate model, providing improved levels of discrimination and confidence. To this end, we applied the PLS-DA model to combine our 43 selected markers to obtain the AUC (Figure 9A), and predicted the classification probability into each chemotype (Figure 9B). The performance of this model was tested using a balanced Monte-Carlo cross-validation procedure, and as a result the average accuracy based on 100 cross-validations was 0.991.

**Figure 10:**
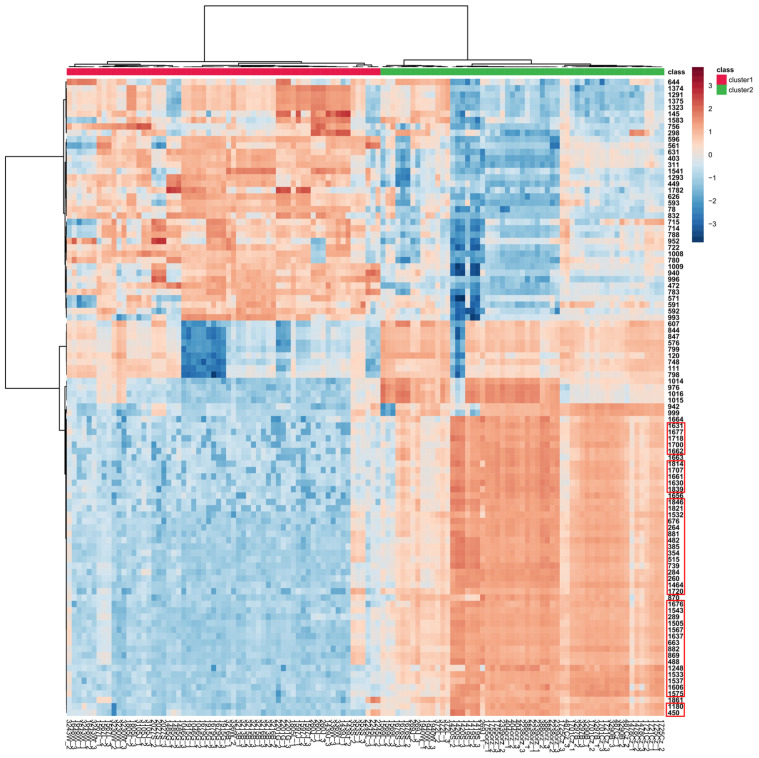
Hierarchical clustering with the heat map generated from the top 100 tentatively identified metabolites present in the tested *Diaporthe* isolates, according to *t*-tests, using Pearson distance for similarity measure and Ward’s linkage algorithm for clustering. Clusters were grouped based on the HCA/*k*–means analyses shown in Figure 6B and Figure 7. Cell colors indicate relative concentration values as high (dark brown) or low (dark blue), with samples in columns and features (MS DIAL ID in NI) in rows. Features from Table 3 are enclosed in red rectangles.

Hierarchical clustering with a heat map is also shown to easily visualize the concentration variation of the top 100 tentatively identified metabolites (according to *t*-tests) expressed in the tested *Diaporthe* isolates (Figure 10). A sharp contrast of their accumulation is observed, while at the same time the samples are clearly grouped by their group membership, determined by HCA and *k*–means analyses.

The study on the utilization of metabolites as chemotaxonomic markers for species identification refers to the genus *Penicillium*, *Aspergillus*, *Fusarium*, *Alternaria*, and the *Xylariaceae* family [75,76]. However, in the case of *Diaporthe*, this type of research was limited. In the research conducted by Horn et al. [77,78] on endophytic *Phomopsis* (=*Diaporthe*) from woody host, three metabolites named phomodiol, phomopsolide B, and phomopsichalasin were indicated as potential chemotaxonomic markers for this fungi. In addition, Abreu et al. [79] showed that the production of secondary metabolites by *Phomopsis* and related *Diaporthales* may be species-specific, indicating the value of utilizing the metabolic analysis in taxonomic research on closely related species.

In our research, isolates belonging to the *Diaporthe eres* species complex isolated from fruit trees produced 153 metabolites from which 43 were recognized as potential chemotaxonomic markers, mostly belonging to the drimane sesquiterpenoid—phthalide hybrid class. This group included mainly phytotoxic compounds such as cyclopaldic acid and altiloxin A, B and their derivatives. It is noteworthy that during our investigation, the phytotoxic compound cyclopaldic acid was produced not only by the pathogenic *Diaporthes* species but also by the endophytic *D. eres* isolate 1420S, previously described by Abramczyk et al. [32] and used in the present study. Following the observations of Graniti et al. [67] and McMullin et al. [69], the production of phytotoxic cyclopaldic acid may be related to *Diaporthe* changing its lifestyle from endophytic to pathogenic, under favorable conditions. Thus, it is possible that endophytic *D. eres* isolate 1420S [32], is a weak opportunistic pathogen, switching from an endophytic to a pathogenic phase when the host tissue becomes weakened. This issue requires more advanced research in the future.

## 3. Materials and Methods

### 3.1. Chemicals and Reagents

Hypergrade for LC-MS acetonitrile (≥99.9%) and HPLC gradient-grade methanol (≥99.9%) were purchased from Merck (Darmstadt, Germany), LC-MS grade formic acid (98–100%) was purchased from Sigma Aldrich (Steinheim, Germany). A Milli-Q Simplicity 185 water purification system from Millipore (Milford, MA, USA) was used for preparation of ultrapure water (18.2 MΩ·cm).

### 3.2. Fungal Strains and Culture Conditions

We investigated 40 *Diaporthe* strains isolated during previous studies from different species of fruit trees growing in south-eastern Poland (Table 1) [4,31]. All axenic cultures were deposited at the Fungal Collection of Phytopathology and Mycology Subdepartment, University of Life Sciences in Lublin (Poland). Thirty-nine came from shoots with visible disease symptoms and one from healthy *Prunus domestica* as endophyte, described previously by Abramczyk et al. [32]. *Diaporthe* strains were isolated according to the methodology described by Król [80]. Healthy fragments of the tested plants were properly disinfected by rinsing several times, first in a 10% sodium hypochlorite solution, then in sterile distilled water. After drying, the plant fragments were placed on potato dextrose agar (PDA, Difco) and incubated for 5 days at 25°C, in the dark. When the fungus colonies appeared, pure cultures were prepared according to the methodology described previously [80].

### 3.3. DNA Extraction, Amplification and Sequencing

Strains were incubated on PDA at 25 °C for 7 days before to DNA extraction. The total genomic DNA was extracted using the FastDNA^®^SPIN Kit and the FastPrep^®^Instrument (Qbiogene, Inc., Carlsbad, CA, USA), according to the manufacturer’s protocol. All extracted DNA was stored at −20 °C until use.

The amplification of the fragment of the internal transcribed spacer region (ITS) of the nuclear ribosomal RNA gene, the universal primers ITS1: TCCGTAGGTGAACCTGCGG and ITS4: TCCTCCGCTTATTGATATGC were used [81]. For the amplification of ITS regions, 25 μL of the reaction mixture was prepared, which consisted of the following components: 1 μL of genomic DNA (5 ng/μL), DreamTaq™ Green PCR Master Mix (2×) (Thermo Scientific, Waltham, MA, USA) in a volume of 12.5 μL, primers (10 μM) in a volume of 1 μL each and purified water in a volume of 9.5 μL. The PCR reaction was run under the following conditions: 95 °C for 3 min, followed by 39 cycles of 95 °C for 30 sec, 55 °C for 50 sec, 72 °C for 1 min, and final extension at 72 °C for 10 min. Sequencing of the obtained PCR products was performed in the Genomed S.A. (Warsaw, Poland). The sequence data received were deposited in GenBank (Table 1). The Bionumerics 7.6 (Applied Maths NV., Sint-Martens-Latem, Belgium) and SEED v.2.1.05 (Institute of Microbiology CAS, Prague, Czech Republic) software was used for bioinformatic analyses.

The obtained sequences were blasted against the NCBIs GenBank nucleotide database to determine the closest related species.

### 3.4. Extraction of Fungal Metabolites

For metabolite extraction, 40 *Diaporthe* strains were three-point inoculated on 90 mm Petri plates containing PDA, and incubated for 28 days at 23 °C under a 12 h photoperiod, referring to the methodology of Abreu et al. [80], with modifications. Fungal discs (5-mm diameter) were collected in three individual biological repetitions each (*n* = 3). Each fungal culture (120 total) and three non-inoculated medium samples were freeze-dried (Christ Gamma 1–16 LSC, Martin Christ, Osterode am Harz, Germany) and subsequently ground with a mortar. Dried material (25 mg) was transferred to a 5 mL screw-capped centrifuge tube (Eppendorf, Hamburg, Germany) and added to 2.5 mL of extraction solvent mixture, MeOH/H_2_O 80:20 (*v*/*v*). Samples were then thoroughly vortex-mixed for 1 min and ultrasonicated for 20 min under 4 °C. Samples were centrifuged (18,000× *g* for 20 min under 4 °C), and the supernatants were transferred to separate vials and analyzed using UHPLC-QTOF HRMS. A QC (Quality Control) sample (aliquot of all samples) was also prepared and injected six times before randomized sample injection for column conditioning and at every forty samples to evaluate the performance of the LC-MS method during the detection.

### 3.5. UHPLC-QTOF HRMS Profiling

Ultrahigh-performance liquid chromatography-quadrupole time of flight-high-resolution MS (UHPLC-QTOF HRMS) analyses were performed on an Impact II HD mass spectrometer (Bruker, Billerica, USA) coupled to a U-HPLC Ultimate 3000 RSLC system (Thermo Fisher Scientfic, Hemel Hempstead, UK). Five-microliter injections of samples were fed from a thermostatted autosampler (8 °C) onto a CORTECS T3 column (150 mm × 2.1 mm i.d., 2.7 μm, Waters, Milford, USA), equipped with a guard column, and the column was kept at 35 °C. Mobile phases were (A) ultrapure water with 0.1% formic acid (FA), and (B) acetonitrile with 0.1% FA. The flow rate was set at 500 μL/min and the solvent gradient profile was as follows: 0.0–1.0 min, 5% B; 1.0–27.0 min, 5–99% B (concave-shaped gradient—Dionex gradient curve 6); 27.0–30.0 min, 99% B. Between the injections, the column was equilibrated with six volumes of 5% B. Mass detection was performed using an electrospray source in positive ionization (PI) and negative ionization (NI) modes. Ionization spray voltages were set to 4.0 kV (for PI) and 3.0 kV (for NI); dry gas flow was 6 l/min; the dry gas temperature was 200 °C; collision cell transfer time was 90 μs; and nebulizer pressure was 0.7 bar. MS1 and MS/MS data (range 80–1800 *m/z*) were collected using Bruker DataAnalysis 4.3 software in data-dependent acquisition (DDA) mode—after each full MS1 scan, the two most intense ions were fragmented with collision energies of 20 eV for PI and 30 eV for NI.

### 3.6. Data Processing and Metabolite Identification

LC-MS raw data were first converted into the ‘Analysis Base File’ (ABF) format [82] using Reifycs Abf (Analysis Base File) Converter (https://www.reifycs.com/AbfConverter/ (accessed on 25 May 2021)) and processed with MS-DIAL (RIKEN, version 4.90) [83]. MS1 and MS2 tolerances were set to 0.01 and 0.05 Da, respectively, in centroid mode for each data set (PI and NI). In PI and NI modes, automatic feature detection was performed between 3.0 and 27.0 min for mass range between 80 and 1800 Da. The minimum peak height intensity was set to 2000 for NI and 3000 for PI modes, respectively; linear-weighted moving average as the smoothing method using 5 scans and peak width 5 scans. Peaks were aligned on a QC reference file with an RT tolerance of 0.10 min and a mass tolerance of 0.015 Da and retained in the feature table if they appeared in at least 3 samples. All peaks detected from non-inoculated medium were removed from the generated matrix if their “Sample average/blank average” ratio was lower than 10, thus removing the background and contaminants and preserving the true biological mass signals from LC-MS data.

The kept significant features were exported to the MS-FINDER program (RIKEN, version 3.52) for in silico-based annotation using the hydrogen rearrangement rules (HRR) scoring system [84]. The MS1 and MS2 tolerances were set to 10 and 25 ppm, respectively, and the isotopic ratio tolerance set to 20%. The formulas were filtered to exclusively contain only C, H, O, N, P, S, and Cl atoms. Selected compounds were searched against the built-in database in the MS-FINDER system: NANPDB (Northern African Natural Products Database), KNApSAcK, COCONUT, T3DB (the toxin and toxin target database), and NPA (Natural Products Atlas), and only structures with a score above 5 were retained for thorough analysis. Fungal metabolites were tentatively identified by their high-resolution mass data, MS/MS fragmentation pattern analysis, UV data, and published literature.

### 3.7. Multivariate Statistical Analysis

The aligned data table was LOWESS (locally weighted scatterplot smoothing), normalized using the pooled QC samples and exported from MS-DIAL software to comma-separated value (CSV) format prior to analysis using MetaboAnalyst (version 5.0) [85]. The data were filtered by removing variables showing low repeatability among QC samples (RSD > 20%). Two data matrices were constructed, one in PI mode (120 isolates × 3557 metabolites) and the second in NI mode (120 isolates × 1759 metabolites). The samples were then normalized by the sum to account for the effects of sample dilution (different content of culture medium in the samples), data were log10-transformed to correct for heteroscedasticity and Pareto-scaled to reduce the influence of intense peaks, which transformed the data matrix into a more Gaussian-type distribution [86,87]. First, unsupervised principal component analysis (PCA) was used as an exploratory data analysis to provide an overview of LC-MS fingerprints. Unsupervised groups from the PCA were assigned by k–means clustering analysis and confirmed by hierarchical cluster analysis (HCA) performed to obtain a dendrogram of fungal strains according to metabolite profiling (Pearson distance measure, Ward’s clustering algorithm). On the clusters obtained, a partial least squares discriminant analysis (PLS-DA) was conducted using clusters as Y value, and their potential variables were selected based on variable importance in projection (VIP > 1.0) values and false discovery rate (FDR < 0.05) by Wilcoxon rank-sum test.

## 4. Conclusions

The results of our study demonstrated a rich diversity of metabolites secreted by the tested *Diaporth eres* species complex. The characterization of these compounds could be the basis for the future research on their isolation and bioactive potential for agricultural applications as biopesticides or biofertilizers.

Furthermore, the future research should include a larger population of *Diaporthe* from fruit plants from various areas of Poland. It would be worth determining their metabolic profile, then isolating more important compounds to confirm their structure and bioactive properties. In addition, the optimization of culture media and cultivation conditions for producing richer metabolite profiles are necessary for a more conclusive chemical classification of these fungi.

Although the bioactivity of cyclopaldic acid and altiloxins (the main components of the drimane sesquiterpenoid—phthalide hybrids) identified in the present study as potential biomarkers for species belonging to the *Diaporthe eres* complex is known, as described above, the genes involved in their biosynthesis have not yet been defined. In general, the eukaryotic genes involved in a single metabolic pathway are scattered throughout the genome, whereas the genes required for a fungus to produce a given secondary metabolite are very frequently clustered, adjacent to one another on the chromosome [88]. Such clusters are found in the majority of filamentous fungi and may range from only a few to more than 20 genes [89]. Thus, identifying a biosynthetic gene cluster for the main compounds reported as biomarkers for species from *Diaporthe eres* complex, could be the next step to supplement the current research by the results relied on the genetic methods used on a larger *Diaporthe* population.

Over the last decade, multi-locus DNA sequence data and morphological characterization have been extensively used to identify *Diaporthe* on a species level [3,7,10,20,21,90,91,92]. The gene regions most commonly used for this purpose in *Diaporthe* are the internal transcribed spacer (ITS), together with translation elongation factor-1α (EF-1α), β-tubulin, partial histone H3 (HIS), and calmodulin (CAL) [3,6,20,21,93,94]. However, they are still limited to those species for which the comparative sequence data have been deposited in the public database. Nevertheless, a multi-locus sequencing should always be used for identification of *Diaporthe* species [6]. In agreement with the study of Abreu et al. [79] and Horn et al. [77], the metabolite profiling may support phenotypic species recognition in *Diaporthe.* Thus, when studying closely related species in the *Diaporthe eres* complex, a holistic approach combining morphological characterization, metabolic profile and multi-locus sequencing for species identification is certainly worth considering [79].

Characterizing metabolites biosynthesized by *Diaporthe* infecting shoots of fruit trees is vital for the phytotoxic properties and chemotaxonomy. It is also essential to better understand the conditions under which the fungi start producing the toxins and switch their lifestyle from endophytic to pathogenic.

Finally, it is hoped that the results from our initial research will enrich the biodiversity of the chemical compounds of species from *Diaporthe eres* complex and provide a series of new information for this genus.

## Figures and Tables

**Table 1:** Fungi most closely related to *Diaporthe* based on ITS sequences using BLASTn analysis.

Isolate	Host Plant (Shoot)	GenBank Accession No.	Closest Related Species	Similarity [%]	Coverage [%]
260J	*Malus domestica*	OK474176	*D. eres*_HQ533144	100	99.8
269J		OK474177	*D. eres*_KU712214	100	100
1439J		OK474180	*D. eres*_HQ533144	100	100
1597J		OK474183	*D. eres*_MK352454	100	100
3105J		OK474188	*D. eres*_GQ996572	100	100
1046G	*Pyrus communis*	OK474190	*D. eres*_MK352454	100	100
1485G		OK474193	*D. eres*_HQ533144	100	100
1679G		OK474196	*D. eres*_MK352454	100	100
1915G		OK474198	*D. eres*_MK352454	100	100
2201G		OK474201	*D. eres*_MH931269	100	100
336W	*Prunus cerasus*	OK474203	*D. eres*_EU571099	100	99.8
1648W		OK474204	*D. eres*_EU571099	100	99.8
1940W		OK474205	*D. eres*_MW228360	99.6	100
3230W		OK474214	*D. eres*_EU571099	100	99.8
3243W		OK474216	*D. eres*_KX274026	100	100
353S	*Prunus domestica*	OK474217	*D. eres*_GQ996572	100	100
1419S		OK474220	*D. eres*_EU571099	100	99.8
1420S *		**MW664034**	*D. eres* EU571099	100	99.8
1676S		OK474223	*D. eres*_EU571099	100	100
2027S		OK474225	*D. eres*_EU571099	100	99.8
487CZ	*Prunus avium*	OK474227	*D. eres*_EU571099	100	99.8
1478CZ		OK474228	*D. eres*_EU571099	100	99.8
1701CZ		OK474229	*D. eres*_EU571099	100	99.8
1721CZ		OK474230	*D. eres*_EU571099	100	99.8
1725CZ		OK474231	*D. eres*_EU571099	100	99.8
388ORZ	*Juglans regia*	OK474233	*D. eres*_GQ996572	100	100
404ORZ		OK474234	*D. eres*_KX274026	100	100
1755ORZ		OK474236	*D. eres*_GQ281804	99.8	99.8
2238ORZ		OK474237	*D. eres*_HQ533144	100	99.3
2339ORZ		OK474238	*D. eres*_EU571099	100	100
372L	*Corylus avellana*	OK474239	*D. eres*_HQ533144	100	100
1567L		OK474240	*D. eres*_KX274026	100	100
1569L		OK474241	*D. eres*_MK352454	100	100
1805L		OK474246	*D. eres*_GQ996572	100	100
2245L		OK474247	*D. eres*_EU571099	100	100
3213B	*Prunus persica*	OK474250	*D. eres*_MK352454	99.8	100
3215B		OK474251	*D. eres*_MK352454	99.8	100
3216B		OK474252	*D. eres*_MK352454	99,8	100
3290B		OK474253	*D. eres*_HQ533144	99,8	100
3297B		OK474254	*D. eres*_GQ996572	100	100

* in bold *D. eres* as endophyte [32].

**Table 2:** Annotation of specific metabolites in the studied *Diaporthe* isolates using UHPLC-qTOF-MS/MS in the negative (NI) and positive ionization (PI) modes.

No	MS-DIAL ID (NI)	MS-DIAL ID (PI)	Rt (min)	UV (nm)	Meas. *m*/*z*	[Adduct Type]	Neutral Formula	MW	Error (ppm)	Major Fragments *	Putative Metabolite	Cmp. Class
**1**	289		3.69	215, 262	511.1095	[2M − H]^−^	C_11_H_12_O_7_	256.21	0.74	**255.0510**, 211.0614, 181.0519, 135.0448	Islandic acid-II	Pyranone (α-pyrone)
**2**	120	270	3.79	220	197.0809	[M − H_2_O + H]^+^	C_10_H_14_O_5_	214.22	0.16	197.0807, **179.0704**, 151.0754, 137.0594	Multiplolide A	10-membered lactone
**3**	1349	579	3.98	215, 282	230.1022	[M + NH_3_ + H]^+^	C_10_H_12_O_5_	212.20	0.47	195.0655, 177.0538, **149.0598**	Pyrenocine P	Pyranone (α-pyrone)
**4**	107		4.15	215	423.1293	[2M − H]^−^	C_10_H_12_O_5_	212.20	2.17	211.0611, **167.0698**, 111.0434	4-[5-(1-Hydroxyethyl)furan-2-yl]-4-oxobutanoic acid	γ-keto acid
**5**	1015	2082	4.58	215	332.1707	[M + H]^+^	C_15_H_25_NO_7_	331.36	−0.97	314.1602, **296.1504**, 278.1386, 197.0808, 179.0710, 151.0764	Phomopsolide B derivative tiglic acid amide isomer I	Pyranone or furanone
**6**	108	394	4.70	220, 285	213.0755	[M + H]^+^	C_10_H_12_O_5_	212.20	−0.71	**195.0651**, 177.0546, 167.0703, 149.0595, 139.0393	Scirpyrone K	Pyranone (α-pyrone)
**7**	264		4.86	220	253.0354	[M − H]^−^	C_11_H_10_O_7_	254.19	−0.09	179.0379, **164.0103**	Strobide B (cyclopaldic acid derivative)	Phthalide
**8**	976		4.87	215	657.2882	[2M − H]^−^	C_15_H_23_NO_7_	329.35	−0.04	**328.1409**, 229.0713	Dehydro-phomopsolide B derivative tiglic acid amide	Pyranone or furanone
**9**	174	701	4.99	215, 255	239.0550	[M + H]^+^	C_11_H_10_O_6_	238.19	0.06	221.0443, 203.0335, 193.0499, 177.0545, **175.0391**, 160.0153	Convolvulanic acid A isomer I	Phthalide
**10**	1016	1856	5.10	215	314.1603	[M − H_2_O + H]^+^	C_15_H_25_NO_7_	331.36	−1.47	**314.1599**, 296.1485, 278.1402, 197.0812, 179.0705	Phomopsolide B derivative tiglic acid amide isomer II	Pyranone or furanone
**11**	1014	2087	5.85	215	332.1704	[M + H]^+^	C_15_H_25_NO_7_	331.36	−1.47	314.1598, **296.1495**, 278.1385, 197.0814, 179.0699, 137.0588	Phomopsolide B derivative tiglic acid amide isomer III	Pyranone or furanone
**12**	1403	2841	5.98	220	419.1375	[M − H_2_O + H]^+^	C_18_H_28_O_10_S	436.47	−1.08	**301.0743**, 283.0627, 255.0682, 237.0573, 179.0694	Unidentified	
**13**	173		6.10	220	237.0404	[M − H]^−^	C_11_H_10_O_6_	238.19	0.26	**165.0137**	Convolvulanic acid A isomer II	Phthalide
**14**	1907	2838	6.11	220	419.1372	[M − H_2_O + H]^+^	C_18_H_28_O_10_S	436.47	−0.16	**301.0743**, 283.0627, 255.0682, 237.0573, 179.0694	Unidentified	
**15**	844	2134	6.63	220	334.1862	[M + NH_3_ + H]^+^	C_15_H_24_O_7_	316.35	−0.54	299.1498, **281.1382**, 213.0779, 181.0863	Dihydrohydroxyphomopsolidone B isomer I	Furanone
**16**	847	1905	6.77	220	317.1595	[M + H]^+^	C_15_H_24_O_7_	316.35	−0.07	299.1505, **181.0863**, 153.0909, 137.0593	Dihydrohydroxyphomopsolidone B isomer II	Furanone
**17**	795	1870	7.06	215	315.1438	[M + H]^+^	C_15_H_22_O_7_	314.33	0.09	**179.0704**, 161.0599, 151.0757, 137.0596, 119.0491	Dihydrohydroxyphomopsolide B isomer I	Pyranone
**18**	739	1468	7.10	220	295.0819	[M − H_2_O + H]^+^	C_14_H_16_O_8_	312.27	−2.15	**277.0702**, 249.0749, 221.0445, 193.0485, 161.0601	Isariketide	Polyketide
**19**	482	1042	7.31	220	267.1596	[M − H_2_O + H]^+^	C_15_H_24_O_5_	284.35	−1.81	**249.1469**, 231.1386	Hydroxy-altiloxin A isomer-I	Drimane sesquiterpenoid
**20**	508	1058	7.35	220	269.1024	[M − H_2_O + H]^+^	C_13_H_18_O_7_	286.28	0.93	169.0495, **151.0384**, 123.0441	Unidentified	
**21**	1404	2839	7.41	220	419.1375	[M − H_2_O + H]^+^	C_18_H_28_O_10_S	436.47	−0.62	**301.0746**, 197.0808, 189.0214, 179.0702	Unidentified	
**22**	488	2085	7.51	220	267.1593	[M − H_2_O + H]^+^	C_15_H_24_O_5_	284.35	−0.75	**249.1483**, 231.1374, 205.1586, 189.1277	Hydroxy-altiloxin A isomer II	Drimane sesquiterpenoid
**23**	798	2085	7.69	220	332.1708	[M + NH_3_ + H]^+^	C_15_H_22_O_7_	314.33	−1.34	297.1332, 279.1237, **179.0702**	Dihydrohydroxyphomopsolide B isomer II	Pyranone
**24**	1180		7.96	220	363.0717	[M − H]^−^	C_17_H_16_O_9_	364.30	1.25	229.0768, **220.0343**, 179.038	5-Hydroxymethylasterric acid	Diphenyl ether
**25**	111	128	8.43	220	177.0546	[M − 2 × H_2_O + H]^+^	C_10_H_12_O_5_	212.20	−0.37	177.0546, **149.0595**	(3*R*,4*R*,4a*R*,6*R*)-4,8-Dihydroxy-6,7-epoxy-3,4,4a,5,6,7-hexahydro-1H-2-benzopyran-1-one (isomer)	Isocoumarin
**26**	748	2046	8.68	220	330.1555	[M + NH_3_ + H]^+^	C_15_H_20_O_7_	312.32	−2.47	277.1059, 195.0652, **177.0546**, 135.0448	Dihydrohydroxyphomopsolide A	Pyranone
**27**	143	529	8.73	225, 340	225.0758	[M + H]^+^	C_11_H_12_O_5_	224.21	−0.22	**207.0649**, 163.0751, 147.0437	5,7-Dihydroxy-*O*-methylmellein	Dihydroisocoumarin
**28**	1407	2837	8.85	220	419.1370	[M − H_2_O + H]^+^	C_18_H_28_O_10_S	436.47	0.07	301.0743, 197.0809, **179.0701**	Unidentified	
**29**	1311		8.92	220	410.0912	[M − H]^−^	C_27_H_13_N_3_O_2_	411.41	4.90	-	Unidentified	
**30**	882		9.14	220	319.1322	[M − H]^−^	C_15_H_25_ClO_5_	320.81	−1.33	**283.1553**, 265.1455, 221.1563, 203.1414, 165.0917	Dihydro-hydroxy-altiloxin B isomer-I	Drimane sesquiterpenoid
**31**	145	533	9.21	220, 310	225.1119	[M + H]^+^	C_12_H_16_O_4_	224.25	−0.29	179.1066, **165.0912**, 147.0800	Phomopsinone A	Pyrenocine (α-pyrone)
**32**	799	1872	9.25	220	315.1449	[M + H]^+^	C_15_H_22_O_7_	314.33	−5.00	297.1353, 215.0913, **197.0803**, 179.0698	Dihydrohydroxyphomopsolide B isomer III	Pyranone
**33**	869	1650	9.48	220	301.1204	[M − H_2_O + H]^+^	C_15_H_23_ClO_5_	318.79	−0.90	**283.1100**, 265.0990, 255.1149, 247.1329	Hydroxy-altiloxin B isomer I	Drimane sesquiterpenoid
**34**	1861	3947	9.55	220	728.3152	[M + H]^+^	C_31_H_53_NO_16_S	727.82	0.80	648.3593, **338.2323**, 219.1743, 201.1635	Restricticin derivative	-
**35**	78	175	9.59	220	183.1014	[M − H_2_O + H]^+^	C_10_H_16_O_4_	200.23	1.85	**165.0894**	Stagonolide C/G	Macrolide
**36**	646	895	9.69	220	255.1596	[M − H_2_O − CO + H]^+^	C_15_H_24_O_6_	300.35	−0.38	237.1486, 219.1377, 191.1432, 173.1321, **163.1484**	Arecoic acid A/B isomer I	Sesquiterpene
**37**	607	1893	9.97	220	316.1761	[M + NH_3_ + H]^+^	C_15_H_22_O_6_	298.33	−2.13	299.1465, 281.1374, 201.1512, **181.0859**	Phomopsolidone B	Pyranone
**38**	1205		10.08	220	373.0961	[M − H]^−^	C_25_H_14_N_2_O_2_	374.39	3.9	-	Unidentified	
**39**	1312		10.13	220	825.2562	[2M − H]^−^	C_18_H_23_NO_10_	413.38	1.77	221.0813, **177.0914**	Unidentified	
**40**	279	675	10.20	220	237.1484	[M − H_2_O + H]^+^	C_14_H_22_O_4_	254.32	0.87	**219.1373**, 191.1432, 173.1321, 133.1015	Oblongolide R	Naphthofuran (polyketide)
**41**	515	1063	10.46	220	269.1747	[M − H_2_O + H]^+^	C_15_H_26_O_5_	286.36	0.12	251.1639, **233.1531**, 215.1428, 205.1584, 187.1479, 177.0905	Cytospolide F/Q/M	Nonanolide
**42**	644	892	10.47	220	255.1588	[M − H_2_O − CO + H]^+^	C_15_H_24_O_6_	300.35	0.95	**237.1483**, 219.1379, 191.1428, 173.1329, 163.1481	Arecoic acid A/B isomer II	Sesquiterpene
**43**	250		10.47	220	251.1287	[M − H]^−^	C_14_H_20_O_4_	252.31	−0.07	207.1376, 189.1288, **177.1274**, 175.1116	Oblongolide B/C1/E/N isomer I	Norsesquiterpene γ-lactones
**44**	1248		10.59	220	389.0879	[M − H]^−^	C_19_H_18_O_9_	390.34	−0.24	220.0367, 192.0386, 189.0538, **179.0348**, 149.0242	Cladonioidesin	Depside
**45**		331	10.76	220	207.1014	[M + H]^+^	C_12_H_14_O_3_	206.24	0.83	**189.0910**, 174.0675, 161.0961, 146.0722	Phomochromone A	Chromone
**46**		2985	10.76	220	435.1773	[M + H]^+^	C_26_H_26_O_6_	434.48	5.79	**229.0833**	Prenylcandidusin C	Dibenzofuran
**47**	1063		11.39	220	338.2335	[M − H]^−^	C_19_H_33_NO_4_	339.47	2.4	-	Unidentified	
**48**	252		11.43	220	251.1292	[M − H]^−^	C_14_H_20_O_4_	252.31	−0.86	**189.1290**, 187.1132	Oblongolide B/C1/E/N isomer II	Norsesquiterpene γ-lactones
**49**	576	1202	11.52	220	279.1226	[M − H_2_O + H]^+^	C_15_H_20_O_6_	296.32	0.34	261.1116, **219.1015**, 179.0698, 137.0597	Dihydrophomopsolide A	Pyranone
**50**	870		11.62	220	317.1161	[M − H] ^−^	C_15_H_23_ClO_5_	318.79	0.00	**301.2025**	Hydroxy-altiloxin B isomer II	Drimane sesquiterpenoid
**51**	1606		11.64	220	521.2042	[M − H]^−^	C_26_H_34_O_11_	522.54	−2.61	**283.1554**, 265.1450, 193.0503, 163.0398	Hydroxy-altiloxin A—cyclopolic acid hybrid	Drimane sesquiterpenoid—phthalide hybrid
**52**	450	1307	11.74	220	283.0633	[M + H]^+^	C_13_H_14_O_5_S	282.31	0.61	265.0516, 191.0701, **173.0597**, 158.0358, 145.0645	Amycolachromone E	Chromone
**53**	1299		11.88	220	403.1039	[M − H]^−^	C_20_H_20_O_9_	404.37	−1.10	279.0507, **235.0608**, 220.0358, 163.0409	Unidentified	
**54**	385	871	11.89	220	253.1797	[M − H_2_O + H]^+^	C_15_H_26_O_4_	270.37	0.45	**235.1690**, 217.1590, 189.1639, 151.0756	Dihydro-altiloxin A	Drimane sesquiterpenoid
**55**	881		12.02	220	319.1316	[M − H]^−^	C_15_H_25_ClO_5_	320.81	−1.33	**283.1542**, 265.1466, 247.1318, 185.0803	Dihydro-hydroxy-altiloxin B isomer-II	Drimane sesquiterpenoid
**56**	1008	2071	12.02	220	331.2480	[M + H]^+^	C_18_H_34_O_5_	330.46	−0.30	313.2382, 295.2276, **277.2165**, 259.2058	Trihydroxyoctadecenoic acid isomer I	Fatty acid/oxylipin
**57**	1707		12.18	220	568.1605	[M − H]^−^	C_26_H_32_ClNO_11_	569.99	−2.44	**317.1153**, 281.1381, 263.1287, 250.0345, 236.0203, 206.0449, 191.0228, 174.0153	Hydroxy-altiloxin B—isocyclopaldic acid amide hybrid	Drimane sesquiterpenoid—phthalide hybrid
**58**		676	12.19	220	237.1484	[M + H]^+^	C_14_H_20_O_3_	236.31	0.51	**219.1380**, 201.1276, 191.1427, 173.1321, 163.1480	Oblongolide C/D/H/J/P isomer	Norsesquiterpene γ-lactones
**59**		372	12.37	220	211.1332	[M + H]^+^	C_12_H_18_O_3_	210.27	0.34	**193.1211**	Unidentified	Pyranone
**60**	298		12.40	220, 255, 290, 340	257.0454	[M − H]^−^	C_14_H_10_O_5_	258.23	0.57	**215.0346**, 213.0537, 187.0382, 171.0446, 159.0441	Alternariol	Benzochromenone (coumarin derivative)
**61**	942	2032	12.40	220	329.2330	[M + H]^+^	C_18_H_32_O_5_	328.44	−2.28	311.2225, **293.2117**, 275.2008	Trihydroxyoctadecadienoic acid isomer I	Fatty acid/oxylipin
**62**	552	1799	12.48	220	312.1447	[M + NH_3_ + H]^+^	C_15_H_18_O_6_	294.30	−1.48	195.0647, **177.0546**, 135.0445	Phomopsolide A/C	Dihydropyranone
**63**	1664		12.55	220	552.1652	[M − H]^−^	C_26_H_32_ClNO_10_	553.99	−2.90	**317.1169**, 234.0391, 190.0506, 175.0281	Hydroxy-altiloxin B—deoxy-isocyclopaldic acid amide hybrid	
**64**	1543		12.57	220	493.2457	[M − H]^−^	C_26_H_38_O_9_	494.58	−2.82	**211.0597**, 196.0295, 181.0496, 177.0206, 151.0390	Luminacin E1	Sesquiterpenoids
**65**	354	827	12.60	220	251.1644	[M − H_2_O + H]^+^	C_15_H_24_O_4_	268.35	−0.11	**233.1530**, 205.1593, 187.1488, 145.1006	Altiloxin A	Drimane sesquiterpenoid
**66**	1700		12.69	220	566.1454	[M − H]^−^	C_26_H_30_ClNO_11_	567.97	−3.42	**317.1166**, 301.1204, 281.1374, 248.0193, 204.0305	Hydroxy-altiloxin B—dehydro-isocyclopaldic acid amide hybrid	Drimane sesquiterpenoid—phthalide hybrid
**67**	952	1775	12.84	220	311.2223	[M − H_2_O + H]^+^	C_18_H_32_O_5_	328.44	−1.57	**293.2118**, 275.2001	Trihydroxyoctadecadienoic acid isomer II	Fatty acid/oxylipin
**68**	894	1981	12.99	220	324.2174	[M + H]^+^	C_18_H_29_NO_4_	323.43	−3.60	306.2070, **288.1961**	Bipolamide A	Triene amide
**69**	1009		13.03	220	329.2334	[M − H]^−^	C_18_H_34_O_5_	330.46	−0.16	229.1441, **211.1338**, 183.1394, 171.1047	Trihydroxyoctadecenoic acid isomer II	Fatty acid/oxylipin
**70**	1846		13.07	220	680.2120	[M − H]^−^	C_32_H_40_ClNO_13_	682.11	−0.67	**318.0985**, 317.1156, 303.0737, 281.1389, 274.1086, 259.0844, 246.1130, 231.0902	Hydroxy-altiloxin B—methyl-salfredin C3 hybrid	Drimane sesquiterpenoid—phthalide hybrid
**71**	1830		13.12	220	329.2327	[M − H]^−^	C_18_H_34_O_5_	330.46	2.00	229.1443, **211.1331**, 183.1394, 171.1016	Trihydroxyoctadecenoic acid isomer III	Fatty acid/oxylipin
**72**	676	1060	13.14	220	303.1365	[M − H]^−^	C_15_H_25_ClO_4_	304.81	1.19	267.1605, **249.1500**, 223.1693, 141.0918	Dihydro-altiloxin B	Drimane sesquiterpenoid
**73**	1583	3423	13.21	220	514.3136	[M + H]^+^	C_25_H_43_N_3_O_8_	513.63	−2.55	**496.3022**, 452.2766, 382.2589, 364.2488	Arbumycin	Cyclic peptide
**74**	999		13.31	220	329.2328	[M − H]^−^	C_18_H_34_O_5_	330.46	0.75	293.2133, 201.1118, **171.1022**, 139.1113	Trihydroxyoctadecenoic acid isomer IV	Fatty acid/oxylipin
**75**	1254		13.44	220	391.1398	[M − H]^−^	C_20_H_24_O_8_	392.40	−1.00	-	Unidentified	
**76**	1676	3507	13.63	220	555.1639	[M − H]^−^	C_26_H_33_ClO_11_	556.99	−0.07	**317.1157**, 281.1380, 263.1278, 237.0376, 191.0350, 175.0379	Hydroxy-altiloxin B—cyclopolic acid hybrid isomer-I	Drimane sesquiterpenoid—phthalide hybrid
**77**	663	1341	13.70	220	285.1259	[M − H_2_O + H]^+^	C_15_H_23_ClO_4_	302.79	−2.32	**267.1146**, 239.1203, 203.1422, 175.1484	Altiloxin B	Drimane sesquiterpenoid
**78**	780		13.78	220	311.2219	[M − H]^−^	C_18_H_32_O_4_	312.44	−0.70	293.2119, **249.1863**, 231.1748, 157.0865	Dihydroxyoctadecadienoic acid isomer I	Fatty acid/oxylipin
**79**	1821	3804	13.82	220	652.2162	[M − H]^−^	C_31_H_40_ClNO_12_	654.10	0.65	334.0927, **317.1156**, 290.1035, 275.0792, 231.0908, 190.0529	Hydroxy-altiloxin B—dihydro-salfredin A7 hybrid	Drimane sesquiterpenoid—phthalide hybrid
**80**	1046		13.91	220	335.0825	[M − H]^−^	C_16_H_17_ClN_2_O_4_	336.77	−4.90	-	Unidentified	-
**81**	1833		14.01	220	659.4739	[2M − H]^−^	C_18_H_34_O_5_	330.46	0.94	311.2218, 293.2122, 211.1324, **199.1340**	Trihydroxyoctadecenoic acid isomer V	Fatty acid/oxylipin
**82**	940		14.13	220	655.4417	[2M − H]^−^	C_18_H_32_O_5_	328.44	2.32	309.2066, 291.1962, 227.1285, **209.1176**, 197.1180, 185.1179	Trihydroxyoctadecadienoic acid isomer III	Fatty acid/oxylipin
**83**	996		14.21	220	329.2336	[M − H]^−^	C_18_H_34_O_5_	330.46	1.05	293.2116, 211.1356, **199.1345**, 171.1022	Trihydroxyoctadecenoic acid isomer VI	Fatty acid/oxylipin
**84**		221	14.47	215, 320	193.0856	[M + H]^+^	C_11_H_12_O_3_	192.21	1.67	**175.0754**, 147.0805, 132.0577	5-Methylmellein	Benzopyran
**85**	993		14.54	220	329.2328	[M − H]^−^	C_18_H_34_O_5_	330.46	1.36	311.2215, 293.2091, 211.1334, **199.1335**, 181.1232, 169.1221	Trihydroxyoctadecenoic acid isomer VII	Fatty acid/oxylipin
**86**	299		14.64	220, 290	515.1241	[2M − H]^−^	C_12_H_15_ClO_4_	258.70	1.83	**213.0685**, 183.0586	Acremonisol A	Dihydroisocoumarin (aromatic pentaketide)
**87**	1677		14.69	220	555.1662	[M − H]^−^	C_26_H_33_ClO_11_	556.99	−4.20	**317.1154**, 299.1059, 237.0396, 191.0339, 175.0391	Hydroxy-altiloxin B—cyclopolic acid hybrid isomer-II	Drimane sesquiterpenoid—phthalide hybrid
**88**	1612		14.70	220	523.2178	[M − H]^−^	C_26_H_36_O_11_	524.56	−1.17	**285.1711**, 267.1604, 241.1815, 237.0396, 223.1692, 193.0506	Dihydro-hydroxy-altiloxinA—cyclopolic acid hybrid	Drimane sesquiterpenoid—phthalide hybrid
**89**	828		14.71	220	313.2383	[M − H]^−^	C_18_H_34_O_4_	314.46	−0.85	**295.2275**, 277.2166, 259.2035, 235.2090, 157.0861	Dihydroxyoctadecenoic acid isomer I	Fatty acid/oxylipin
**90**	1663		14.73	220	552.1649	[M − H]^−^	C_26_H_32_ClNO_10_	553.99	−1.27	**301.1210**, 250.0360, 206.0481, 191.0226	Altiloxin B—isocyclopaldic acid amide hybrid isomer-I	Drimane sesquiterpenoid—phthalide hybrid
**91**	1662		14.82	220	552.1644	[M − H]^−^	C_26_H_32_ClNO_10_	553.99	−0.37	**301.1207**, 250.0352, 206.0463, 191.0226	Altiloxin B—isocyclopaldic acid amide hybrid isomer-II	Drimane sesquiterpenoid—phthalide hybrid
**92**	178	711	14.88	220	239.1645	[M + H]^+^	C_14_H_22_O_3_	238.32	−1.38	221.1529, 193.1580, **175.1481**, 135.1176, 119.0851	Penihydrone	Cyclic alcohol
**93**	1720		14.95	220	583.1603	[M − H]^−^	C_27_H_33_ClO_12_	585.00	−2.61	**317.1161**, 281.1405, 263.1272, 221.0447, 189.0188, 167.1104	Hydroxy-altiloxin B—*O*-methylisocyclopaldic acid hybrid	Drimane sesquiterpenoid—phthalide hybrid
**94**	1785		14.98	220	311.2227	[M − H]^−^	C_18_H_32_O_4_	312.44	0.27	293.2108, 275.2017, 249.1856, 235.1706, **195.1390**	Dihydroxyoctadecadienoic acid isomer II	Fatty acid/oxylipin
**95**	1661	845	15.43	220	552.1644	[M − H]^−^	C_26_H_32_ClNO_10_	553.99	−0.37	**301.1218**, 250.0359, 206.0457, 191.0226	Altiloxin B—isocyclopaldic acid amide hybrid isomer-III	Drimane sesquiterpenoid—phthalide hybrid
**96**	722	1784	15.53	220	311.2224	[M + H]^+^	C_18_H_30_O_4_	310.43	−2.30	**293.2109**, 275.2007, 187.1115, 159.1152	Hydroxyoxooctadecadienoic acid isomer I	Fatty acid/oxylipin in
**97**	1532		15.54	220	489.2122	[M − H]^−^	C_26_H_34_O_9_	490.54	1.65	**211.0614**, 209.0445, 195.0280, 193.0501, 181.0505, 151.0398	Austalide O	Meroterpenoid
**98**	1718	3602	15.54	220	580.1950	[M − H]^−^	C_28_H_36_ClNO_10_	582.04	0.86	**301.1209**, 278.0668, 263.0430, 247.1330	Altiloxin B—*O*-dimethylisocyclopaldic acid amide hybrid	Drimane sesquiterpenoid—phthalide hybrid
**99**	1505	700	15.69	220	477.2493	[M − H]^−^	C_26_H_38_O_8_	478.58	0.19	403.2497, **211.0607**, 181.0491, 151.0384	Antroquinonol U	Meroterpenoid
**100**		2684	15.79	220	404.2065	[M + NH_3_ + H]^+^	C_22_H_26_O_6_	386.44	0.68	**267.1229**, 233.0818, 147.0650, 129.0551	Colletofragarone A1	Cyclohexenone
**101**	1630	3501	15.82	220	536.1675	[M − H]^−^	C_26_H_32_ClNO_9_	537.99	−0.59	**301.1206**, 234.0417, 191.0452, 175.0275	Altiloxin B—deoxy-isocyclopaldic acid amide hybrid isomer-I	Drimane sesquiterpenoid—phthalide hybrid
**102**	260		15.84	220	251.1648	[M − H]^−^	C_15_H_24_O_3_	252.35	0.27	**207.1738**	Deoxy-altiloxin A	Drimane sesquiterpenoid
**103**	1575		16.01	220	507.2226	[M − H]^−^	C_26_H_36_O_10_	508.56	1.91	**269.1762**, 251.1647, 223.1736, 193.0507, 163.0384	Dihydro-altiloxin A—cyclopolic acid hybrid	Drimane sesquiterpenoid—phthalide hybrid
**104**	284		16.01	220	253.1809	[M − H]^−^	C_15_H_26_O_3_	254.37	0.07	**235.1704**, 209.1891, 193.1591, 177.1280	(Deoxy-dihydro-altiloxin A) Diaporol I	Drimane sesquiterpenoid
**105**		1421	16.04	220	293.2112	[M − H_2_O + H]^+^	C_18_H_30_O_4_	310.43	−0.25	**275.2002**, 219.1386, 179.1453,	Hydroxyoxooctadecadienoic acid isomer II	Fatty acid/oxylipin
**106**	1656		16.07	220	550.1492	[M − H]^−^	C_26_H_30_ClNO_10_	551.97	−1.18	**301.1219**, 283.1122, 265.1450, 248.0206, 176.0324	Altiloxin B—dehydro-isocyclopaldic acid amide hybrid	Drimane sesquiterpenoid—phthalide hybrid
**107**	1839	3843	16.10	220	664.2158	[M − H]^−^	C_32_H_40_ClNO_12_	666.11	1.24	**318.0981**, 301.1211, 303.0744, 274.1095, 259.0842, 246.1126, 231.0901	Altiloxin B—methyl-Salfredin C3 hybrid	Drimane sesquiterpenoid—phthalide hybrid
**108**	714	1422	16.17	220	293.2113	[M − H_2_O + H]^+^	C_18_H_30_O_4_	310.43	−0.58	**275.2004**, 215.1781, 175.1494, 161.1325	Hydroxyoxooctadecadienoic acid isomer III	Fatty acid/oxylipin
**109**	1567	3318	16.19	220	505.2075	[M − H]^−^	C_26_H_34_O_10_	506.54	0.83	**267.1603**, 249.1496, 223.1703, 193.0513, 163.0392	Altiloxin A—cyclopolic acid hybrid	Drimane sesquiterpenoid—phthalide hybrid
**110**	1782		16.40	220	295.2269	[M − H_2_O + H]^+^	C_18_H_32_O_4_	312.44	−0.41	**277.2162**, 259.2048, 161.1326	Dihydroxyoctadecadienoic acid isomer III	Fatty acid/oxylipin
**111**	1374	2961	16.43	220	433.2588	[M + H]^+^	C_25_H_36_O_6_	432.55	−0.77	415.2468, 387.2535, 369.2420, **341.2481**, 285.1835, 239.1797	Wortmannilactone B/D isomer I	Macrolide
**112**	1631	3502	16.44	220	536.1703	[M − H]^−^	C_26_H_32_ClNO_9_	537.99	−1.89	301.1225, **190.0499**, 162.0564	Altiloxin B—deoxy-isocyclopaldic acid amide hybrid isomer-II	Drimane sesquiterpenoid—phthalide hybrid
**113**	1814	3741	16.74	220	636.2213	[M − H]^−^	C_31_H_40_ClNO_11_	638.10	0.65	334.0929, **301.1205**, 290.1025, 275.0799, 231.0904, 190.0511	Altiloxin B—dihydro-salfredin A7 hybrid	Drimane sesquiterpenoid—phthalide hybrid
**114**	1637	3461	16.78	220	539.1686	[M − H]^−^	C_26_H_33_ClO_10_	540.99	0.65	**301.1213**, 265.1457, 221.1567, 193.0505, 175.0396, 163.0394	Altiloxin B—cyclopolic acid hybrid	Drimane sesquiterpenoid—phthalide hybrid
**115**	1669	3460	17.24	220	553.1849	[M − H]^−^	C_27_H_35_ClO_10_	555.01	−0.54	**301.1214**, 283.1080, 207.0668, 175.0394, 147.0429	Altiloxin B—*O*-methylcyclopolic acid hybrid isomer I	Drimane sesquiterpenoid—phthalide hybrid
**116**	788	1512	17.34	220	295.2272	[M − H_2_O + H]^+^	C_18_H_32_O_4_	312.44	−1.37	**277.2156**, 235.2046, 217.1958, 163.1476	Dihydroxyoctadecadienoic acid isomer IV	Fatty acid/oxylipin
**117**	1668	3459	17.37	220	553.1848	[M − H]^−^	C_27_H_35_ClO_10_	555.01	−0.36	**301.1202**, 283.1101, 207.0664, 175.0398	Altiloxin B—*O*-methylcyclopolic acid hybrid isomer II	Drimane sesquiterpenoid—phthalide hybrid
**118**	832	1577	17.61	220	297.2429	[M − H_2_O + H]^+^	C_18_H_34_O_4_	314.46	−1.52	**279.2307**, 261.2215, 167.1051	Dihydroxyoctadecenoic acid isomer II	Fatty acid/oxylipin
**119**		2792	17.79	220	415.2115	[M + H]^+^	C_24_H_30_O_6_	414.49	0.04	281.1393, 135.0814, **119.0854**	4-*O*-methylmelleolide	Sesquiterpene
**120**	715	1758	17.79	220	311.2214	[M + H]^+^	C_18_H_30_O_4_	310.43	0.92	293.2119, 275.2013, **249.2216**, 177.1276	Gallicynoic acid D (Dihydroxyoctadecenynoic acid isomer)	Fatty acid/oxylipin
**121**	783	1526	18.12	220	295.2272	[M − H_2_O + H]^+^	C_18_H_32_O_4_	312.44	−2.65	**277.2161**, 259.2038	Dihydroxyoctadecadienoic acid isomer V	Fatty acid/oxylipin
**122**	1703		18.43	220	567.1631	[M − H]^−^	C_27_H_33_ClO_11_	569.00	−0.90	**317.1164**, 281.1396, 263.1289, 219.1380, 153.0906	Hydroxy-altiloxin B—*O*-methylcyclopaldic acid hybrid	Drimane sesquiterpenoid—phthalide hybrid
**123**	1375	2960	18.68	220	433.2582	[M + H]^+^	C_25_H_36_O_6_	432.55	0.61	415.2480, 387.2521, 369.2433, **341.2478**, 295.2427, 239.1790	Wortmannilactone B/D isomer II	Macrolide
**124**	1291	2676	18.83	220	403.2477	[M + H]^+^	C_24_H_34_O_5_	402.52	0.50	385.2376, 367.2280, 357.2441, **339.2318**, 321.2214, 311.2386, 283.1698, 237.1647	Macrolactin G/I/K isomer	Macrolide
**125**	775	1504	19.02	220	295.2271	[M − H_2_O + H]^+^	C_18_H_32_O_4_	312.44	−1.05	**277.2171**, 167.1430	Dihydroxyoctadecadienoic acid isomer VI	Fatty acid/oxylipin
**126**	1533	3248	19.05	220	489.2134	[M − H]^−^	C_26_H_34_O_9_	490.54	−0.80	**251.1649**, 237.0400, 191.0350, 163.0390	Deoxy-altiloxin A—cyclopolic acid hybrid	Drimane sesquiterpenoid—phthalide hybrid
**127**	1537		19.26	220	491.2297	[M − H]^−^	C_26_H_36_O_9_	492.56	−2.12	**253.1811**, 235.1703, 191.0360, 163.0401	Deoxy-dihydro-altiloxin A—cyclopolic acid hybrid (Diaporol I—cyclopolic acid hybrid)	Drimane sesquiterpenoid—phthalide hybrid
**128**	756	1837	19.27	220	313.2381	[M + H]^+^	C_18_H_32_O_4_	312.44	−1.81	295.2274, **277.2167**, 249.2212, 185.1311, 125.0962	Dihydroxyoctadecadienoic acid isomer VII	Fatty acid/oxylipin
**129**	1323	2824	19.48	220	417.2644	[M + H]^+^	C_25_H_36_O_5_	416.55	−0.60	**399.2534**, 371.2595, 353.2478, 325.2533, 297.2572, 239.1786, 197.1328	Macrolactin M	Macrolide
**130**	561		19.67	220	587.4315	[2M − H]^−^	C_18_H_30_O_3_	294.43	1.29	**275.2015**, 195.1361	Oxooctadecadienoic acid I	Fatty acid/oxylipin
**131**		3451	19.79	220	520.3404	[M + H]^+^	C_33_H_45_NO_4_	519.72	3.34	502.3311, 337.2739, 258.1101, **184.0733**	Sespendole	Indolosesquiterpene
**132**	593	1222	20.23	220	279.2324	[M − H_2_O + H]^+^	C_18_H_32_O_3_	296.45	1.88	261.2233, 237.1848, **209.1537**, 195.1388, 181.1222	Hydroxyoctadecadienoic acid isomer I	Fatty acid/oxylipin
**133**	1464	3051	20.28	220	459.2383	[M − H]^−^	C_26_H_36_O_7_	460.56	1.15	385.2379, 379.2274, 357.2444, **195.0297**	Tropolactone D	Meroterpenoid
**134**	596		20.75	220	295.2276	[M − H]^−^	C_18_H_32_O_3_	296.45	0.91	**277.2169**	Hydroxyoctadecadienoic acid isomer II	Fatty acid/oxylipin
**135**	1657		21.26	220	551.1692	[M − H]^−^	C_27_H_33_ClO_10_	553.00	−0.46	**301.1206**, 283.1098, 221.1540	Altiloxin B—*O*-methylcyclopaldic acid hybrid (Pestalotiopen A)	Drimane sesquiterpenoid—phthalide hybrid
**136**	560	1491	21.35	220	295.2268	[M + H]^+^	C_18_H_30_O_3_	294.43	−0.10	**277.2170**, 235.1703, 179.1434	Hydroxyoctadecatrienoic acid isomer II	Fatty acid/oxylipin
**137**	1757		21.44	220	612.3671	[M + FA - H]^−^	C_33_H_49_N_3_O_5_	567.76	−2.95	228.0640, **168.0426**, 122.9844, 93.5690	Unidentified	-
**138**	571	1487	21.58	220	295.2266	[M + H]^+^	C_18_H_30_O_3_	294.43	0.58	**277.2157**, 241.1954, 221.1525, 179.1431	Hydroxyoctadecatrienoic acid isomer III	Fatty acid/oxylipin
**139**	626		21.59	220	297.243	[M − H]^−^	C_18_H_34_O_3_	298.46	1.74	**279.2327**	Hydroxyoctadecenoic acid isomer I	Fatty acid/oxylipin
**140**	592	1230	22.24	220	279.2322	[M − H_2_O + H]^+^	C_18_H_32_O_3_	296.45	0.53	261.2233, **149.0231**	Hydroxyoctadecadienoic acid isomer III	Fatty acid/oxylipin
**141**	591	1587	22.61	220	297.2432	[M + H]^+^	C_18_H_32_O_3_	296.45	−2.63	279.2305, 251.2366, **183.1373**, 169.1578,	Hydroxyoctadecadienoic acid isomer IV	Fatty acid/oxylipin
**142**	1541		22.61	220	491.3375	[M + FA - H]^−^	C_28_H_46_O_4_	446.66	0.70	427.3211, **425.3062**, 409.3098, 407.2971, 391.2990, 281.2488	Stoloniferone N	Ergostane steroid
**143**	598	1572	22.87	220	297.2426	[M + H]^+^	C_18_H_32_O_3_	296.45	−0.60	279.2305, 251.2366, **183.1373**, 169.1578, 141.1270	Hydroxyoctadecadienoic acid isomer V	Fatty acid/oxylipin
**144**		4070	22.90	220	865.4796	[M + H]^+^	C_55_H_64_N_2_O_7_	865.11	−1.12	459.2595, **389.2176**, 371.2069, 303.1442, 233.1020	Unidentified	
**145**	311		22.96	220	257.2119	[M − H]^−^	C_15_H_30_O_3_	258.40	1.23	**211.2077**, 207.1727, 189.1644	Hydroxypentadecanoic acid	Fatty acid/oxylipin
**146**		4078	23.12	220	865.4789	[M + H]^+^	C_55_H_64_N_2_O_7_	865.11	0.30	459.2589, **389.2171**, 371.2066, 303.1441, 233.1017	Unidentified	-
**147**	602	1565	23.49	220	297.2424	[M + H]^+^	C_18_H_32_O_3_	296.45	−0.60	279.2319, 261.2209, 243.2110, **233.2265**, 167.1431, 135.1176	Hydroxyoctadecadienoic acid isomer VI	Fatty acid/oxylipin
**148**	403		24.15	220	271.2277	[M − H]^−^	C_16_H_32_O_3_	272.42	0.62	**225.2225**, 223.2062, 197.1894	Hydroxyhexadecanoic acid isomer	Fatty acid/oxylipin
**149**	631		24.81	220	297.2433	[M − H]^−^	C_18_H_34_O_3_	298.46	0.73	**251.2377**, 249.2236	Hydroxyoctadecenoic acid isomer II	
**150**		2637	24.91	220	395.3313	[M + H]^+^	C_28_H_42_O	394.63	−1.16	**377.3208**, 311.2371, 293.2264, 251.1790, 211.1486, 157.1013	Ergosta-5,7,9(11),22-tetraen-3beta-ol	Sterol
**151**	449	1291	25.58	220	281.2471	[M + H]^+^	C_18_H_32_O_2_	280.45	1.45	**263.2371**, 245.2259, 161.1332	(Linoleic acid) octadecadienoic acid isomer	Fatty acid
**152**	1293	2391	25.70	220	357.2997	[M + H]^+^	C_21_H_40_O_4_	356.54	0.66	339.2895, 283.2635, **265.2525**, 247.2422	2,3-Dihydroxypropyl oleate (octadecenoyl)-sn-glycerol)	Monoacylglycerol
**153**	472	1329	26.65	220	283.2635	[M + H]^+^	C_18_H_34_O_2_	282.46	−1.22	265.2527, **247.2426**, 163.1482	Octadecenoic acid isomer	Fatty acid/oxylipin

* Numbers in bold represent the base peak.

**Table 3:** The 43 top ranked features contributing to the group discrimination in PLS-DA and marked as potential biomarkers for the tested *Diaporthe* isolates from data generated in NI mode.

No.	MS-DIAL ID	Tentative Identification	VIP Score	FDR Adj. *p*-Value	Fold Change (Cluster 2/Cluster 1)	AUC
**109**	1567	Altiloxin A—cyclopolic acid hybrid	2.20	3.33 × 10^−19^	128	0.995
**33**	869	Hydroxy-altiloxin B isomer I	2.15	5.51 × 10^−19^	51	0.989
**114**	1637	Altiloxin B—cyclopolic acid hybrid	2.14	3.23 × 10^−19^	181	0.998
**126**	1533	Deoxy-altiloxin A—cyclopolic acid hybrid	2.12	3.23 × 10^−19^	283	0.999
**1**	289	Islandic acid-II	2.08	3.23 × 10^−19^	159	0.998
**77**	663	Altiloxin B	2.07	3.54 × 10^−19^	121	0.994
**76**	1676	Hydroxy-altiloxin B—cyclopolic acid hybrid isomer-I	2.05	3.23 × 10^−19^	178	0.996
**133**	1464	Tropolactone D	2.03	3.54 × 10^−19^	264	0.994
**93**	1720	Hydroxy-altiloxin B—*O*-methylisocyclopaldic acid hybrid	2.01	4.71 × 10^−18^	19	0.972
**99**	1505	Antroquinonol U	2.00	3.54 × 10^−19^	96	0.994
**95**	1661	Altiloxin B—isocyclopaldic acid amide hybrid isomer-III	1.99	1.73 × 10^−18^	348	0.979
**41**	515	Cytospolide F/Q/M	1.97	1.04 × 10^−18^	72	0.983
**72**	676	Dihydro-altiloxin B	1.96	3.23 × 10^−19^	138	0.996
**54**	385	Dihydro-altiloxin A	1.95	4.83 × 10^−19^	93	0.991
**22**	488	Hydroxy-altiloxin A isomer-II	1.94	7.42 × 10^−19^	53	0.986
**18**	739	Isariketide	1.93	3.23 × 10^−19^	236	0.997
**64**	1543	Luminacin E1	1.93	3.23 × 10^−19^	59	0.998
**79**	1821	Hydroxy-altiloxin B—dihydro-salfredin A7 hybrid	1.93	3.33 × 10^−19^	143	0.995
**113**	1814	Altiloxin B—dihydro-salfredin A7 hybrid	1.93	3.23 × 10^−19^	266	0.996
**24**	1180	5-Hydroxymethylasterric acid	1.92	3.23 × 10^−19^	131	0.999
**70**	1846	Hydroxy-altiloxin B—methyl-salfredin C3 hybrid	1.92	3.23 × 10^−19^	174	0.996
**98**	1718	Altiloxin B—*O*-dimethylisocyclopaldic acid amide hybrid	1.92	7.85 × 10^−19^	217	0.986
**65**	354	Altiloxin A	1.91	5.51 × 10^−19^	82	0.989
**30**	882	Dihydro-hydroxy-altiloxin B isomer-I	1.91	5.34 × 10^−19^	54	0.989
**57**	1707	Hydroxy-altiloxin B—isocyclopaldic acid amide hybrid	1.91	3.23 × 10^−19^	165	0.997
**7**	264	Strobide B	1.91	3.23 × 10^−19^	95	0.997
**102**	260	Deoxy-altiloxin A	1.90	3.23 × 10^−19^	135	0.999
**127**	1537	Diaporol I—cyclopolic acid hybrid	1.90	3.23 × 10^−19^	114	0.998
**44**	1248	Cladonioidesin	1.88	3.23 × 10^−19^	150	1.000
**19**	482	Hydroxy-altiloxin A isomer-I	1.87	8.10 × 10^−19^	84	0.985
**104**	284	Diaporol I	1.87	3.23 × 10^−19^	98	0.999
**51**	1606	Hydroxy-altiloxin A—cyclopolic acid hybrid	1.86	3.23 × 10^−19^	153	0.998
**91**	1662	Altiloxin B—isocyclopaldic acid amide hybrid isomer-II	1.86	1.06 × 10^−18^	146	0.983
**101**	1630	Altiloxin B—deoxy-isocyclopaldic acid amide hybrid isomer-I	1.86	7.65 × 10^−19^	192	0.986
**55**	881	Dihydro-hydroxy-altiloxin B isomer-II	1.85	9.38 × 10^−19^	56	0.984
**107**	1839	Altiloxin B—methyl-Salfredin C3 hybrid	1.84	9.22 × 10^−19^	207	0.984
**87**	1677	Hydroxy-altiloxin B—cyclopolic acid hybrid isomer-II	1.84	1.06 × 10^−18^	78	0.983
**103**	1575	Dihydro-altiloxin A—cyclopolic acid hybrid	1.83	3.54 × 10^−19^	90	0.994
**112**	1631	Altiloxin B—deoxy-isocyclopaldic acid amide hybrid isomer-II	1.83	6.66 × 10^−19^	115	0.987
**97**	1532	Austalide O	1.82	3.67 × 10^−19^	42	0.993
**52**	450	Amycolachromone E	1.82	1.61 × 10^−18^	40	0.980
**66**	1700	Hydroxy-altiloxin B—dehydro-isocyclopaldic acid amide hybrid	1.81	3.23 × 10^−19^	103	0.996
**80**	1046	Unidentified	1.81	5.51 × 10^−19^	73	0.989

## Data Availability

The data presented in this study are openly available in Zenodo at https://doi.org/10.5281/zenodo.7371706 (accessed on 28 November 2022).
